# Epitheloid hemangioendothelioma of urinary bladder

**DOI:** 10.4103/0970-1591.40624

**Published:** 2008

**Authors:** Narmada P. Gupta, Surendra B. Kolla, Sabyasachi Panda, M. C. Sharma

**Affiliations:** Department of Pathology, All India Institute of Medical Sciences, New Delhi, India

**Keywords:** Hemangioendothelioma, urinary bladder

## Abstract

Epitheloid hemangioendothelioma is an uncommon vascular neoplasm and has an unpredictable clinical behavior. It is characterized by round or spindle-shaped endothelial cells with cytoplasmic vacuolation. Most often, epitheloid hemangioendothelioma arise from the soft tissues of the upper and lower extremities and it has borderline malignant potential. We describe the first reported case of epitheloid hemangioendothelioma in the urinary bladder, which was treated by transurethral resection. The diagnosis was confirmed by immunohistochemistry.

## INTRODUCTION

Vascular tumors are uncommon and show a wide clinicopathological variation ranging from benign to borderline malignant to complete malignant lesions. Epitheloid vascular neoplasms are a rare group of tumors characterized morphologically by the presence of epitheloid endothelial cells. Vascular tumors composed of these cells are further divided into three distinct varieties: epitheloid hemangioma, epitheloid hemangioendothelioma (EHE), and epitheloid angiosarcoma.[1] Hemangioendotheliomas are vascular tumors of low-malignant potential with a behavior intermediate between hemangioma and conventional angiosarcoma.[1] Epitheloid hemangioendothelioma is an uncommon vascular neoplasm and has an unpredictable clinical behavior.

Vascular tumors like cavernous hemangiomas and few cases of angiosarcoma have been reported to occur in the urinary bladder.[2] However, to the best of our knowledge, till date there is no documentation of the occurrence of EHE in the urinary bladder. We describe the first case of EHE of the urinary bladder which was confirmed by immunohistochemistry (IHC).

## CASE REPORT

A 17-year-old male patient presented with history of episodic painless hematuria of 7-year duration and obstructive voiding symptoms with overflow incontinence for 2 years. There was no history of any bowel symptoms or neurological deficit in the lower half of the body. Physical examination revealed a palpable distended bladder. A firm mass was palpable above the prostate on digital rectal examination.

This patient had his first episode of hematuria 7 years prior to his presentation to our hospital. At that time he was evaluated in another hospital where a computerized tomographic scan (CECT) of the abdomen was done which revealed a 2.4 × 2.5 × 2.3 cm^3^ mass at the bladder base. Multiple biopsies were done at different centers and were reported as cystitis, histiocytic neoplasm, inflammatory myofibroblastic tumor, and rhabdomyosarcoma. The IHC was not done at any point of time for pathological diagnosis. The patient never received definitive treatment before presenting to us with the above-mentioned complaints.

**Figure 1:**
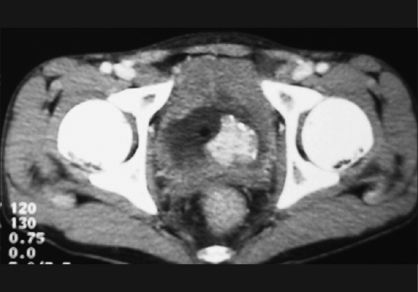
CECT of the abdomen showing bright enhancing tumor over the trigone with calcification

This patient had normal renal parameters (blood urea and serum creatinine) at presentation. Urine cytology was negative for malignant cells. Owing to incontinence and bilateral hydroureteronephrosis (HDUN), per urethral catheterization was done and subsequently imaging studies were performed. Ultrasound (U/S) of the abdomen revealed 4 × 4.3 × 4.3 cm^3^ mass in the trigone with a thickened urinary bladder and bilateral HDUN. Micturition cystourethrogram revealed elongated diverticulated bladder (mimicking neurogenic bladder) with no vesicoureteric reflux and high-postvoid residual urine (PVR). Magnetic resonance imaging of the spine was normal. The CECT of the abdomen revealed a 4-cm solid enhancing mass over the base of the bladder with no perivesical extension or lymphadenopathy [Figure 1]. Cystoscopy revealed a solid polypoidal mass over the trigone with an incomplete calcified rim and a normal prostate. A transurethral complete resection of the tumor was done and deep biopsies were taken. Tumor was highly vascular and bleeding during resection. The patient had uneventful postoperative course and voided normally with insignificant PVR after the catheter was removed.

**Figure 2:**
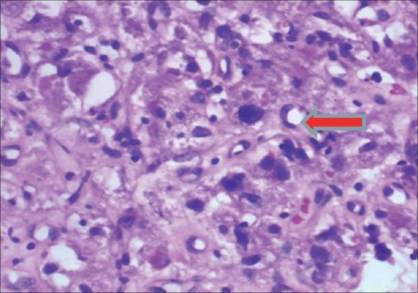
Red arrow indicates intracytoplasmic vaculization (H and E ×400

**Figure 3:**
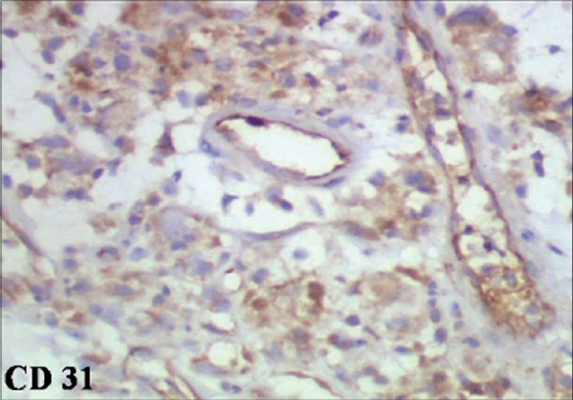
Immunohistochemical staining with CD 31

Pathological examination revealed polygonal to spindle-shaped tumor cells with abundant eosinophilic cytoplasm and at places showing intracellular vacuolization [Figure 2]. Mucin staining with Alcian blue-Periodic acid Schiff (PAS) was negative excluding the possibility of mucin in the intracytoplasmic vesicle. There was marked nuclear pleomorphism and prominent nucleoli and occasional mitoses were identified. The IHC for desmin, CD-34, CD-68, S-100, epithelial membrane antigen (EMA), chromogranin, and cytokeratin were negative but positive for endothelial cell markers CD-31 [Figure 3] and Factor VIII. The overall features were suggestive of epitheloid hemangioendothelioma. At 1-year follow-up, the patient was asymptomatic and ultrasound showed no recurrence in the bladder, no HDUN, and an insignificant PVR. Cystoscopy showed no tumor recurrence in the bladder.

## DISCUSSION

The EHE was first described by Weiss and Enzinger in 1982 as a borderline or low-grade neoplasm.[3] Owing to overlapping histological features, these tumors are considered a spectrum of lesions, where EHE is believed to be in the middle of the spectrum with a behavior intermediate between epitheloid hemangioma and epitheloid angiosarcoma. While EHE occurs most commonly in the soft tissue of the extremeties, visceral involvement (bone, liver, spleen, lung, etc.) has also been reported. In the present case, the young age of the patient, the absence of any conditions that might predispose to transitional-cell carcinoma, as well as the fact that the tumor has not progressed significantly over 7 years despite not offering definitive treatment has made us believe that the tumor could be a benign non-urothelial tumor.

The differential diagnoses that were considered based on the pathological findings are shown in Table 1. Characteristic histopathologic features of EHE include: plump cells with eosinophilic hyaline cytoplasm, angiocentric location, cytoplasmic vacuoles representing primitive vascular lumina, cells arranged in cords, a chondromyxoid matrix, and papillary tufts of plump cells within lymphovascular spaces.[1] Confirmatory evidence is described as erythrocytes within cytoplasmic vacuoles or primitive tumor-cell lined channels which mirrors the primitive vasoformative character of EHE and immunohistochemical evidence of endothelial differentiation.[1] It has been widely accepted that CD31 is the most specific and sensitive endothelial marker.[4] Use of multiple vascular markers (CD31, Factor VIII related antigen, CD34, Ulex europeus) is useful in questionable cases.

**Table 1:** Differential diagnosis of spindle cell tumors of urinary bladder

Rhabdomyosarcoma
Paraganglioma
Granular cell tumor
Inflammatory myofibroblastic tumor
Epitheloid hemangioendothelioma
Epitheloid leiomyosarcoma
Postoperative spindle cell tumor

It is commonly held that EHE represents a low-grade malignant or borderline vascular neoplasm and in the World Health Organization (WHO) classification of soft-tissue tumors it has been placed under the heading of “intermediate endothelial tumors”.[5] Due to its malignant potential a thorough clinical investigation to search for metastases at the initial diagnosis, as well as for recurrent disease during follow-up can be recommended. The EHE shows variable clinical behavior, prognostication is difficult to correlate with histologic appearance and no reliable prognostic factors have been established. Wide local excision is the most commonly recommended treatment option for EHE. However, in the present case, owing to the indolent behavior of the tumor for a long duration and the tumor being located over the trigone, we attempted transurethral complete resection of the tumor. The completeness of resection was confirmed by taking deep biopsies which were negative. The patient had no recurrence at 1-year of follow-up.

This case is the first of its kind to report the occurrence of EHE in the urinary bladder and also highlights the importance of immunohistochemical diagnosis for identifying such rare non-urothelial bladder tumors. Owing to the rarity and varying histologic appearance of vascular neoplasms, examination by more than one pathologist should be advocated.
